# Biomechanical evaluation of self-cinching stitch techniques in rotator cuff repair: The single-loop and double-loop knot stitches

**DOI:** 10.1515/med-2021-0211

**Published:** 2021-02-12

**Authors:** Stephan Frosch, Gottfried Buchhorn, Fabian Kück, Tim Alexander Walde, Wolfgang Lehmann, Christopher Spering

**Affiliations:** Department of Trauma Surgery, Orthopaedics and Plastic Surgery, University Medical Center Göttingen, Robert-Koch-Strasse 40, 37075, Goettingen, Germany; Department of Medical Statistics, University Medical Center Göttingen, Humboldtallee 32, 37073 Goettingen, Germany

**Keywords:** rotator cuff repair, suture techniques, tendon repair, cyclic loading, ultimate load

## Abstract

In rotator cuff repair, strong and reliable suturing is necessary to decrease failure rates. The biomechanics of two self-cinching stitches – the single-loop knot stitch (SLKS) and the double-loop knot stitch (DLKS) – and the modified Mason-Allen stitch (mMAS) were compared. Twenty-seven porcine infraspinatus tendons were randomized among the three stitches. Each was cyclically loaded (10–80–200 N for 50 cycles each) while the gap formation was measured. Next, ultimate load to failure was tested. The gap widths after cyclic loading were 8.72 ± 0.93 mm for the DLKS, 8.65 ± 1.33 mm for the mMAS, and 9.14 ± 0.89 mm for the SLKS, without significant differences. The DLKS showed the highest ultimate load (350.52 ± 38.54 N) compared with the mMAS (320.88 ± 53.29 N; *p* = 0.304) and the SLKS (290.54 ± 60.51 N; *p* < 0.05). The DLKS showed similar reliability and better strength compared with the mMAS, while the SLKS showed a slight but not significant decrease in performance. In our experience, the DLKS and SLKS have clinical advantages, as they are easy to perform and the self-cinching loop knot allows the surgeon to grasp degenerative tendon tissue. Initial intraoperative tightening of the suture complex (preloading) before locking is important in order to decrease postoperative elongation.

## 1 Introduction

The rotator cuff tear is one of the most common shoulder injuries causing pain and shoulder dysfunction [1,2,3]. Restoration of full rotator cuff integrity is the aim of surgical repair in order to reduce pain and improve shoulder function [4]. Early failure after rotator cuff repair is the most common complication and rerupture rates of 15–94% of chronic, massive rotator cuff tears are reported [1,3,4,5,6,7]. The risk of a rerupture is multifactorial, depending on tear size and thickness peculiarity, age of the patient, and repair technique [3].

Arthroscopic as well as mini-open procedures are common in rotator cuff repair. Arthroscopic repair techniques have become popular in recent years, with possible advantages in visualization of tears and additional intra-articular lesions, less scar formation, and shorter postoperative recovery [4]. On the other hand, arthroscopic repair can be technically demanding and time-consuming in comparison with mini-open procedures [8]. The modified Mason-Allen stitch (mMAS) technique is common in mini-open procedures and considered to be superior to the simple or mattress stitch with respect to initial fixation strength [9,10]. Furthermore, the mMAS shows similar biomechanical and clinical results when compared with double-row fixation [9,10,11]. Rotator cuff failures often occur during the early postoperative stage, while the integrity of the suture mostly depends on the fixation of the suture–tendon interface [12,13]. Therefore, techniques that create strong and reliable sutures are required. In a previous biomechanical cadaver study, the double-loop knot stitch (DLKS) showed superior ultimate-load-to-failure strength when compared with the mMAS (382.2 vs 309.3 N; *p* < 0.05) [14]. Especially in mini-open procedures, where space for the use of a round needle under the acromion is limited, the horizontal stitch configuration of the loop in the single-loop knot stitch (SLKS) and DLKS makes repairs relatively easy to perform compared with the bulky vertical stitch of the mMAS. The self-cinching loop knot of the SLKS and DLKS enhances transverse compression of the tendon tissue as axial strain increases. This effect allows a more effective grasping of frayed tendon tissue and enables the surgeon to grab smaller parts of the tendon without losing the slipping resistance of the suture.

Cyclic loading, rather than ultimate-load-to-failure testing, simulates repetitive loading of the tendon in the early postoperative stage. In order to examine the repetitive load resistance of the SLKS and DLKS in comparison with the mMAS, we performed a cyclic loading program using harvested porcine infraspinatus tendons. It was hypothesized that the DLKS and the SLKS would yield better or at least equal results in cyclic loading compared with the mMAS.

## 2 Materials and methods

### 2.1 Sample preparation

Twenty-seven porcine shoulders were harvested from corpses of Göttingen minipigs (female adult pigs of similar weight and age) and stored at about −38°C. The animals had been sacrificed for a previous unrelated experiment, and the research related to animals use has been complied with all the relevant national regulations and institutional policies for the care and use of animals. The shoulders were thawed at room temperature 10 h before preparation. The infraspinatus muscle and tendon were exposed with care and dissected from the protruding scapula crista. The tendon was then cut sharply, directly from its bony insertion at the tuberculum of the humerus. The latter was inspected for regular anatomy and discarded. All tendons were roughly 25–30 mm long and had a cross-section of approximately 15 × 6 mm. The preparations were then randomly allocated to three groups of nine samples each. In each group, one of the three suture configurations was tested. The testing began immediately after tendon preparation.

### 2.2 Suture

A high-strength, multistrand polyethylene suture, Fiber-Wire No. 2 (Arthrex, Karlsfeld/München, Germany), was taken from a reel and combined with a round, sharpened solitary needle. The sutures were placed at a distance of 15 mm from the end of a tendon. Care was taken to always use comparably placed and sized portions of the tendon. The width and thickness of the tendon in the plane of the sutures were measured. To avoid the need for an additional knot to anchor the ends of the threads, a custom-made compensator device was designed. By means of adjustable deflection rollers, the branches of the thread were oriented parallel to the direction of tension. The thread ends were then each clamped to a branch of the axially centred compensator to allow for length compensation in the case of single-sided suture lengthening. This allowed both ends of the suture to be equally loaded.

**Figure 1:**
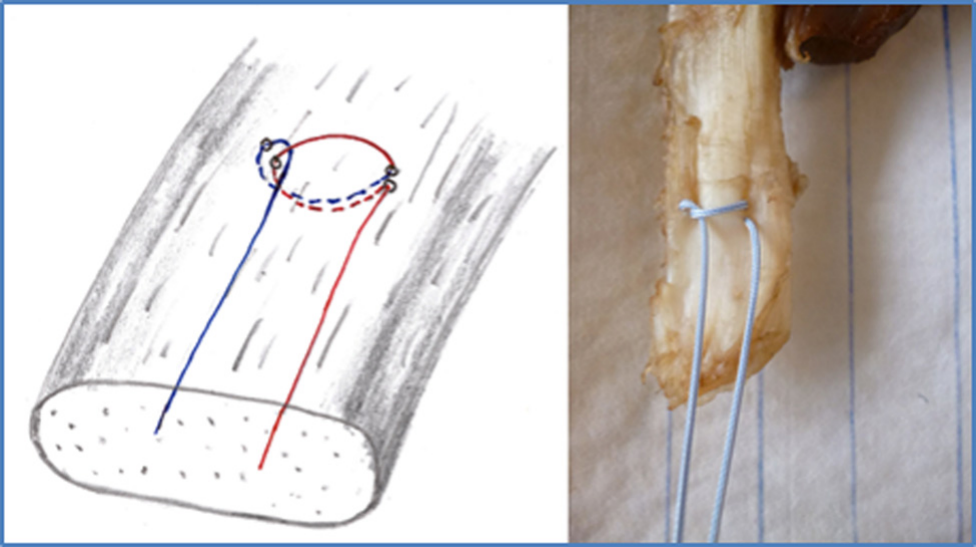
Single-loop knot stitch (SLKS). Schematic illustration on the left. The photo on the right shows that only a smaller part of the tendon is grasped with the SLKS.

**Figure 2:**
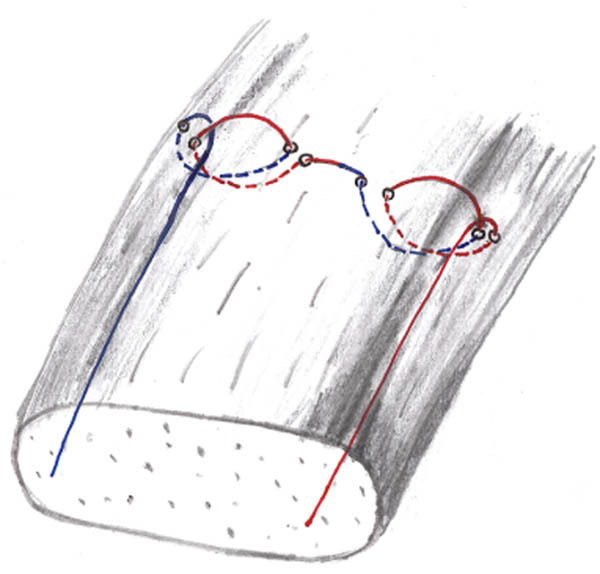
Double-loop knot stitch (DLKS), made of two consecutive SLKS.

**Figure 3:**
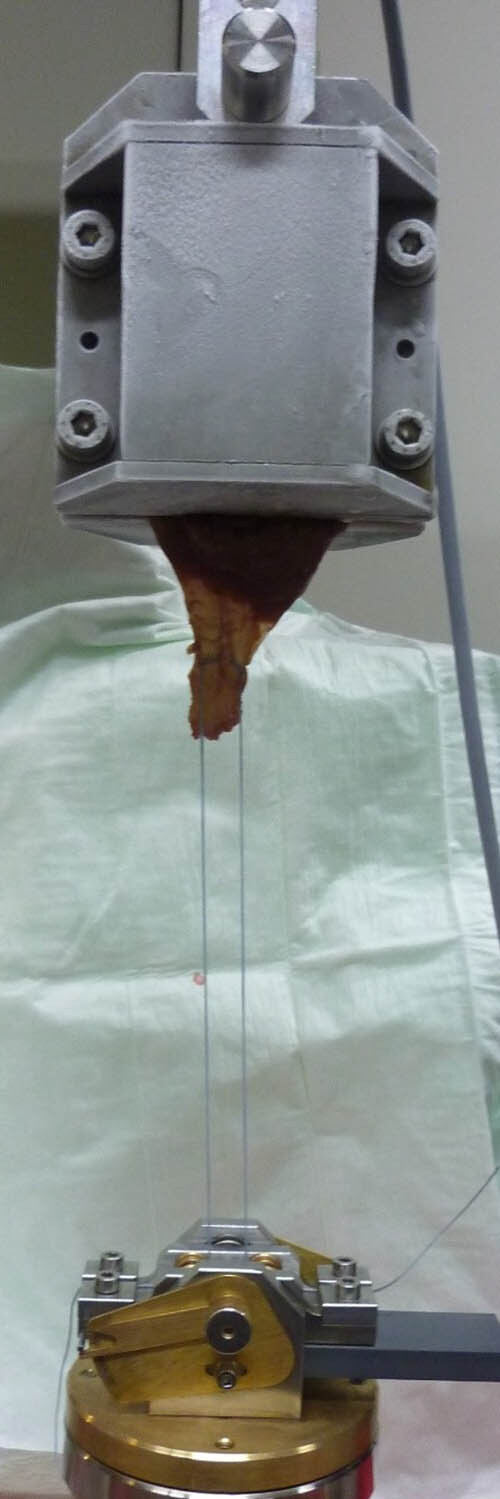
Part of the Zwick UTM test setup. DLKS is shown. The infraspinatus muscle is clamped using two metal brackets of a cryo-jaw (upper part of the picture). The compensator device is mounted on the UTM base (lower part of the picture).

Three suture techniques were tested as follows:

1. The mMAS technique [15].
2. The SLKS, requiring two horizontal passes through the tissue to form a self-cinching sling with a knot that tightens continuously as tension on the thread increases (Figure 1) [14]. Care was taken to not completely penetrate the tendon but rather to only encompass the upper, bursal portion of the cross-section.
3. The DLKS, made of two consecutive single-loop knot stitches, is created using a single thread (Figures 2 and 3) [14]. This stitch is performed using the same techniques as the SLKS, but with a mirrored second stitch.

### 2.3 Biomechanical testing

The specimens were subjected to cyclic loading and ultimate load to failure using a Zwick 1446 universal testing machine (UTM) (Zwick-Roell AG, Ulm, Germany). The fixation protocol described by Baums et al. was used [16]. In short, the infraspinatus muscle was clamped in compression using the two metal brackets of a cryo-jaw (Figure 3). Each bracket had three transverse recesses, 5 mm deep, to be filled with muscle tissue under compression. To achieve reliable fixation and to prevent slippage of the muscle, the metal bags of the brackets were filled with pellets of dry ice to freezing the protuberances and prevent slippage. Care was taken to freeze only the clamped part of the muscle, while the downward-protruding tendon and suture remained unaffected. The cryo-jaw was attached to the load cell and crossbar of the UTM with a cardan joint. The compensator device was mounted on the UTM base in order to load the two threads equally (Figure 3). The data were recorded using testing software (textX-pert V 112.1, Zwick-Roell AG, Ulm, Germany). The elongation (precision = 0.5 mm) and load (precision = 0.1 N) were measured and displayed as a load/elongation curve. The maximum possible error of transverse movement was 0.05%. The calibrated force transducer (maximum load 500 N) had an accuracy of 1% with values above 200 N.

After pre-tension to 40 N, the prepared specimens were cyclically loaded at a displacement rate of 1 mm/s. The cyclic loading started at 10–80 N for 50 cycles and was gradually increased by 20 N every 50 cycles (10–100 N, 10–120 N, etc.) until it reached 10–200 N. After 50 cycles at 10–200 N, the ultimate load to failure was tested. The failure of the ultimate load (*F*_max_ [N]) testing was defined as 20% loss of the ultimate tensile strength independent of failure mode (suture thread cutting through the tendon or breaking of the suture thread).

### 2.4 Statistical analysis

The distribution of gap formation (mm) and *F*_max_ (N) were described by their mean ± standard deviation. The mean was first calculated per animal in order to have just one representative value per animal within each category. Gap formation was visualized separately for each method and force level.

In order to account for the dependencies within the same animal, linear mixed effects models were used with the method, force level, and their interaction as fixed effects for the gap formation and method as fixed effect for the ultimate load. General linear hypothesis testing was carried out for the method comparisons within each force level.

The significance level was set to *α* = 5% for all statistical tests. All analyses were performed with the statistical programming environment R (version 3.4.0, www.r-project.org).

## 3 Results

**Figure 4:**
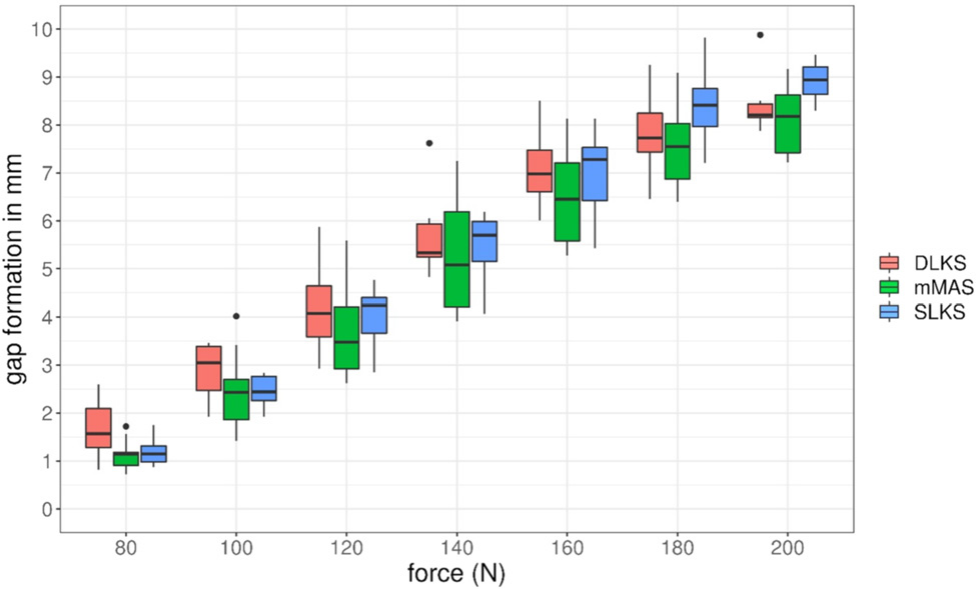
Gap formation in cyclic loading (10 to 80–200 N) of the three types of stitches: DLKS (brown), SLKS (blue), and mMAS (green).

In cyclic loading, the DLKS and mMAS showed comparable gap formation results (8.72 ± 0.93 mm vs 8.65 ± 1.33 mm, *p* = 1) after 350 cycles (Tables 1 and 2; Figure 4). The gap formation of the SLKS was somewhat higher (9.14 ± 0.89 mm) but not significantly different than the DLKS (*p* = 0.26) or the mMAS (*p* = 0.32) (Table 1; Figure 4).

**Table 1:** Mean ± standard deviation of gap formation in cyclic loading (10 to 80–200 N) and *F*_max_ (N) in ultimate load testing within each stitch technique

	Force (N)	DLKS	mMAS	SLKS
Gap formation (mm)	80	1.68 ± 0.62	1.15 ± 0.32	1.19 ± 0.3
	100	2.88 ± 0.62	2.45 ± 0.83	2.46 ± 0.36
	120	4.19 ± 0.99	3.71 ± 1	4 ± 0.7
	140	5.73 ± 0.92	5.19 ± 1.17	5.46 ± 0.79
	160	7.09 ± 0.83	6.44 ± 1.03	6.96 ± 1.03
	180	7.83 ± 0.91	7.56 ± 0.99	8.42 ± 0.89
	200	8.72 ± 0.93	8.65 ± 1.33	9.38 ± 1.14
*F*_max_ (N) (ultimate load to failure)		350.52 ± 38.54	320.88 ± 53.29	290.54 ± 60.51

**Table 2:** *p* value of pairwise comparison of the gap formation in cyclic loading of the DLKS, SLKS, and mMAS

Comparison (N)	*p* value
DLKS.80 – mMA.80	0.9316
DLKS.80 – SLKS.80	0.9843
mMA.80 – SLKS.80	1
DLKS.100 – mMA.100	0.9697
DLKS.100 – SLKS.100	0.9884
mMA.100 – SLKS.100	1
DLKS.120 – mMA.120	0.9575
DLKS.120 – SLKS.120	1
mMA.120 – SLKS.120	0.9938
DLKS.140 – mMA.140	0.961
DLKS.140 – SLKS.140	1
mMA.140 – SLKS.140	0.9979
DLKS.160 – mMA.160	0.7872
DLKS.160 – SLKS.160	1
mMA.160 – SLKS.160	0.6429
DLKS.180 – mMA.180	1
DLKS.180 – SLKS.180	0.5395
mMA.180 – SLKS.180	0.242
DLKS.200 – mMA.200	1
DLKS.200 – SLKS.200	0.2644
mMA.200 – SLKS.200	0.3225

Each DLKS and mMAS suture–tendon complex survived 350 cycles. One SLKS suture–tendon complex did not survive the 10–160 N loading and one did not survive the 10–180 N loading due to suture cutting-out.

The DLKS showed the highest ultimate load with a mean value of 350 N (±38.54), compared with the mMAS (320.88 ± 53.29 N; *p* = 0.304) and the SLKS (290.54 ± 60.51 N; *p* < 0.05), while the only significant difference was between the DLKS and SLKS (*p* < 0.05) (Table 1).

## 4 Discussion

The most important finding of this study is that the DLKS and to a lesser extent the SLKS showed excellent biomechanical behaviour under cyclic loading conditions when compared with the mMAS as the gold standard in (mini) open rotator cuff repair.

The arthroscopic treatment of chronic, massive rotator cuff tears can be technically demanding, with longer operative times, higher costs, and possible increased rerupture rates compared with open treatment [8,17]. Therefore, open and mini-open procedures are still common and frequently used in rotator cuff repair [8]. Both the SLKS and DLKS are applicable to arthroscopic technique, but less technically demanding in open repair. The advantages of the loop–knot technique are most effective in chronic, massive tears with degenerative and/or frayed tendon tissue. The self-cinching property of the loop knot enhances tissue grip as axial strain increases and enables the surgeon to grasp frayed tendon tissue more effectively. The loop knot enhances transverse compaction of the tendon fibres and thereby increases resistance against axial cutting of the thread through parallel-running fibre sheaths of the tendon.

The findings of Ponce et al. in their biomechanical study support our understanding of the beneficial effect induced by transverse compaction of the tendon [18]. They compared (among other stitch techniques) the biomechanical properties of three self-cinching stitches. The configuration of the lasso-mattress stitch induces considerable transverse compaction of the tendon tissue as axial strain is applied. In contrast, the configuration of the self-cinching lasso-loop and the double-cinch stitch induces more axial compression of the threads, parallel to the fibre sheaths of the tendon, as axial strain increases. Consequently, the lasso-mattress stitch bore superior ultimate loads compared with the lasso-loop stitch (148.1 vs 64.7 N) and double-cinch stitch (148.1 vs 97.1 N). Furthermore, the lasso-loop stitch showed superior results in ultimate loading conditions when compared with the mMAS (148.1 vs 128.3 N) and simple stitches such as the mattress stitch (148.1 vs 67.1 N) and the simple stitch (148.1 vs 47.1 N). The authors concluded that self-cinching stitches lead to superior tissue-holding strength in comparison with other comparable noncinching simple stitches. These findings are consistent with our results, as the DLKS showed superior results compared with the mMAS (345.56 vs 320.88 N in ultimate load), and with a previous study, where the DLKS showed significantly superior values compared with the mMAS (382.2 vs 309.3 N; *p* = 0.038) [14]. The forces survived by the SLKS in ultimate load testing were insignificantly lower than those survived by the mMAS. However, it is notable that the amount of tendon tissue grasped by the SLKS was considerably less than that grasped by the mMAS, which might explain these findings.

Cyclic loading, rather than ultimate load-to-failure configurations, simulates repetitive loading of the tendon during the early stages of rehabilitation. The results from cyclic loading did not significantly differ among the DLKS, SLKS, and mMAS. All three suture configurations reached 120 N in cyclic loading before gap formation exceeded 5 mm. Notably, gap formation exceeding 5 mm is considered a clinically relevant failure of the suture–tendon complex. Force analysis of the rotator cuff predicts forces acting on the supraspinatus from 60 N during basic elevation of the arm, up to 117 N with maximal isometric abduction, and of 175–353 N with maximal concentric elevation of the arm [19,20,21,22]. However, the results of the present biomechanical study cannot be directly applied to clinical treatment. From our results, we conclude that the DLKS, SLKS, and mMAS are suitable for passive mobilization in the early postoperative phase, but active mobilization could overstrain the suture–tendon complex over time.

Lorbach et al. examined the single-row modified Mason-Allen stitch in a biomechanical laboratory study using porcine infraspinatus tendons [23]. The cyclic loading of the specimens started at 20 N for 50 cycles, increasing stepwise by 20 N until it reached 200 N for 50 cycles. Only the results for 100, 160, and 200 N were reported. The mean elongation of the construct was 6.4 mm after 100 N, 9.7 mm after 160 N, and 12.3 mm after 200 N of 50 cycles at each force level. The reported values are somewhat high compared with our results, but the differences between them and our values of about 3–4 mm are consistent throughout different loadings. This might be due to that study’s different preloading of the suture–tendon construct of 10 N compared with 40 N in our study. In progressive cyclic loading, the cinching loop tightens up to a certain extent, which increases the thread length between knot and anchor, leading to additional elongation. Ponce et al. confirmed additional elongation of self-cinching sutures in loading configurations [18]. Therefore, initial intraoperative tightening of the knot (preloading) before locking the stitch is important to decrease postoperative elongation.

It is possible to place two separate DLKSs at the proximal and distal ends of the rupture and tie the opposing threads on each side of the tendon (two knots). Alternatively, both DLKSs can be placed with one continuous thread and one final locking knot. It should be noted that tightening two consecutive DLKSs with one thread is more difficult because of the self-cinching mechanism.

One limitation of this study is that the results of an *in vitro* animal study cannot be directly translated to suture techniques for the rotator cuff in human patients. However, the mechanical properties of pig infraspinatus tendons are considered comparable with those of human tendons and are similar to human rotator cuff tendons in size, shape, histological parameters, and mechanical properties [24,25]. Furthermore, the present *in vitro* animal model is frequently used in the literature and allows for easy comparison of results. The enhanced transverse compression force on the tendon encompassed by the suture raises concerns of local tendon necrosis. Theoretically, larger tendon cross-sections better withstand compression forces of constriction. Gerber et al. demonstrated for the mMAS that these forces do not cause long-term histological changes within the tendon and that they are biologically tolerated [26]. Further histologic investigations regarding self-cinching stitches are necessary.
